# In Situ Ruminal Digestion, Fermentation Parameters, and Forage Nutritive Value of Cool-Season Baleage Ensiled under Contrasting Inoculant Strategies

**DOI:** 10.3390/ani12212929

**Published:** 2022-10-25

**Authors:** Sarah Lynn Shoup, Russell Brian Muntifering, Mary Kimberly Mullenix, Liliane Severino Silva, Sandra Leanne Dillard

**Affiliations:** 1 Department of Animal Sciences, Auburn University, Auburn, AL 36849, USA; 2 Department of Animal and Veterinary Sciences, Clemson University, Blackville, SC 29817, USA

**Keywords:** cool-season forages, fermentation parameters, in situ dry matter, microbial inoculation, baleage

## Abstract

**Simple Summary:**

In southeastern USA, the most common method of preserving forages for the winter months is employing dry hay, but spring rains can make it difficult to dry high-moisture forages without there being large dry matter losses. Baleage is a high-moisture feed that is similar to silage that can offer more flexibility for forage producers. However, little is known about the forage quality of different cool-season annual forage mixtures when they are harvested and stored as baleage. A study was conducted to determine the nutritive value and ruminal digestion of cool-season annual mixtures that are ensiled as baleage with and without silage inoculants. Baleage was sampled at 0, 7, 14, 21, 28, 45, 60, and 120 d and it was analyzed for nutritive value and fermentation parameters. Wheat + brassica ensiled at the lowest pH, while the annual ryegrass + oats + crimson clover did not reach a pH that was below 5.0. The DM disappearance of all of the mixtures was greatest in the first 12 h. The effectiveness of the silage inoculant used was inconsistent across the forage treatments. There is no advantage from the use of silage inoculant in these cool-season annual mixtures; however, all of the mixtures had a sufficient nutritive value to meet the nutrition requirements of growing beef cattle.

**Abstract:**

In southeastern USA, the use of baleage has increased as an alternative technology to hay production, thereby allowing for a timelier harvest of the conserved forage. A series of studies were conducted to determine the nutritive value, fermentation parameters, and in situ disappearance of the cool-season annual forage mixtures that were ensiled with or without silage inoculant for up to 120 d. The forage mixtures were wheat (*Triticum aestivum* L.) + brassica hybrid (*Brassica rapa* L. × *napus* L.) (WB), wheat + crimson clover (*Trifolium incarnatum* L.) (WC), and annual ryegrass (*Lolium multiflorum* Lam.) + oat (*Avena sativa* L.) + crimson clover (ROC). The inoculant strategy affected the CP concentration (*p* < 0.05), with it increasing in WB and decreasing in ROC. Among the mixtures, the DM concentration decreased by up to 5%, and the NDF and ADF concentrations decreased by up to 10% during the ensiling period. The pH averaged 5.0, 5.0, and 5.5 for the WC, WB, and ROC mixtures, respectively. Based on our results, the baleage of the cool-season annual forage mixtures may provide a viable high-quality option to sustain animal growth and performance.

## 1. Introduction

In southeastern USA, cool-season annual forages are often used as conserved feeds due to their high nutritive value [1,2]. Forage species adapted to the Gulf Coast region of the US include annual ryegrass (*Lolium multiflorum* Lam.), wheat (*Triticum aestivum* L.), oat (*Avena sativa* L.), triticale (× *Triticosecale Wittm*. Ex A. Camus [*Secale* × *Triticum*]), and clovers (*Trifolium* spp.). These cool-season annuals fill the gap in the seasonal forage availability in warm-season perennial forage systems such as bahiagrass (*Paspalum notaum* Flueggé) and bermudagrass (*Cyndon dactylon* (L.) Pers.). These species are often used in mixtures to optimize the herbage production and nutritive value [1]. The nutritive value of these forage species ranges from 19–45% NDF and 14–25% ADF, with CP values of 18% or greater [1].

The high biomass potential of cool-season annuals often means that surplus forage is produced. This forage can be harvested and stored for periods of slow forage growth (e.g., during winter and later summer). One of the feed preservation methods is baleage, which involves making round bales out of high-moisture forage and then wrapping the bales with oxygen impermeable plastic. This creates an anaerobic environment allowing for fermentation [2]. The use of baleage in the southeastern USA has increased due to frequent rainfall during the spring and summer which makes it difficult to make high-quality hay [3,4]. For baleage production, the moisture levels should be between 40 and 60% at the time of baling to prevent plant respiration and proteolysis [5,6]. The rate of the pH decline is crucial to ensuring that the proper fermentation occurs for high rate of recovery of the DM, energy, and nutrients in the preserved feeds [7]. Microbial inoculants are often used to improve the lactic acid fermentation, decrease the DM losses, and inhibit the deleterious microbes [8]. In a 2-year study, McCormick et al. evaluated the nutritive value of annual ryegrass, which was stored as dry hay, haylage (20 to 40% DM), or baleage, and they reported greater crude protein (CP) concentrations for the baleage (19.8%), which was followed by the haylage (19.2%), and the hay (13.1%) [9]. However, the effect of baleage practice and the use of a microbial inoculant on the nutritive value and dry-matter properties of the preserved baleage has not been systematically examined.

The objective of this study was to determine the nutritive value, fermentation parameters, and in situ dry matter disappearance of three cool-season annual forage mixtures including wheat, brassica hybrid, crimson clover, oat, and annual ryegrass that were ensiled with and without a microbial inoculant for up to 120 days after the ensiling occurred (DAE).

## 2. Materials and Methods

### 2.1. Study Location

**Figure 1:**
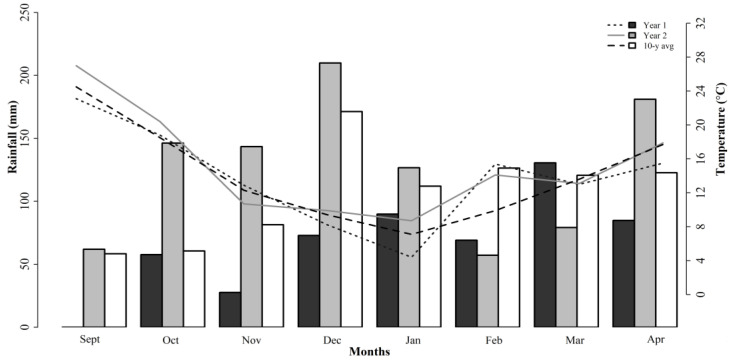
Monthly weather data during studies 1, 2, and 3 and 10-year average at Shorter, AL. Bars and lines indicate cumulative monthly rainfall and average temperatures, respectively.

Three different forage mixtures for the study were planted in the Edwin V. Smith Research Center (EVS) in Shorter, AL (32.3951° N, 85.9184° W). Soils at the site are classified as a Marvyn loamy sand (Typic Kanhapludults (thermic, fine-loamy, kaolinitic)). Soil pH was 6.6 prior to the studies being performed so no liming was required. Potassium (K) and phosphorus (P) fertilizers were applied according to Alabama Cooperative Extension System recommendations after soil testing was performed [10]. The 10-year total rainfall from September to April averaged 853 mm with minimum and maximum temperatures of 7 and 25 °C, respectively. Weather data were collected at the experiment station, and they are reported in Figure 1.

### 2.2. Forage Mixture Establishment

This study used three different cool-season forage mixtures: wheat (*Triticum aestivum* L.) + brassica hybrid (*Brassica rapa* L. × *napus* L.) (WB), wheat + crimson clover (*Trifolium incarnatum* L.) (WC), and annual ryegrass (*Lolium multiflorum* Lam.) + oat (*Avena sativa*) + crimson clover (ROC). From this point forward, WB is referred to as Study 1, WC is referred to as Study 2, and ROC is referred to as Study 3.

For Studies 1 and 2, WC and WB mixtures were planted in 6 ha area each using a no-till drill (Great Plains, Salina, KS, USA) on 6 October 2017. In southeastern USA, most cool-season annual forage production occurrs on fallow row crop fields. In order to reduce erosion and increase soil health parameters, no-till drilling was the preferred method of sowing. Seeding rates for ‘Baldwin’ wheat, ‘Dixie’ crimson clover, and ‘T-Raptor’ brassica hybrid were 66, 9, and 3 kg pure live seed (PLS) ha^−1^, respectively. At sowing, plots were fertilized using a 17-17-17 urea-based granular formulation that provided 67 kg N ha^−1^, 30 kg P ha^−1^ and 42 kg K ha^−1^, respectively. Two additional N-fertilizer applications at a rate of 67 kg N ha^−1^ were applied on 1 December 2017 and 2 February 2018. A split application of N fertilizer was used as this is standard production practice for management of pastures in southeastern USA. Then, plots were harvested to 10 cm stubble height after mixtures achieved approximately 10% bloom for clover and boot stage for wheat and brassica species. For Study 3, ROC mixture was planted on 18 September 2018 in a 16-ha area. Seeding rates were, respectively, 22, 100, and 33 kg PLS ha^−1^ of ‘Marshall’ annual ryegrass, ‘RAM’ oat, and ‘Dixie’ crimson clover. A no-till drill method was used for oat and crimson clover, whereas annual ryegrass was broadcast. No-till sowing was used in order to reflect common practices in the region for forage and row crop producers alike. After sowing, the plot was fertilized with 67 kg N ha^−1^, 30 kg P ha^−1^ and 42 kg K ha^−1^. An additional N fertilization at rate of 67 kg N ha^−1^ was applied on 4 February 2019 based on common production practices in the region. Forage was harvested to 10 cm stubble height once mixture was at the reproductive stage (approximately 10% bloom for clover and boot stage for grass components).

### 2.3. Experimental Design

Three mini-silo studies were conducted using three different forage mixtures as described above. Treatments were common to all of the three studies, and these consisted of the factorial combinations of two microbial inoculant strategies (with (IN) or without (NIN) microbial inoculation) and eight ensiling periods [0, 7, 14, 21, 28, 45, 60, and 120 days after ensiling (DAE)] which were applied to the forage mixtures. A completely randomized design was used with two replicates (mini silo) per ensiling time in each study.

Harvested forage was placed into cloth bags and dried at room temperature (ca. 25 °C) until forage reached 55% dry matter (DM) at the Auburn University Ruminant Nutrition Laboratory. Forage DM concentration was determined by the microwave method [11]. For the IN treatment, Pioneer 11G22 alfalfa/grass/cereal silage inoculant (Johnston, IA, USA) was used which consists of a combination of *Lactobacillus buchneri* and *L. plantarum*. The inoculant was applied following label instructions of 0.1 g inoculant per mL of deionized water, and simulated target application used of 237 mL per ton of forage. Polyvinyl chloride (PVC) mini silos measuring 60 × 10 cm were used for the ensiling trial. Each silo was filled with forage at an average density of 1.86 g cm^−3^. Prior to their use, PVC pipes were fitted with two FERNCO flexible PVC quick-caps (10 cm dia., Davison, MI, USA) and fitted with a one-way gas-release valve and tested for air leakage. Eight time periods were used: 0, 7, 14, 21, 28, 45, 60, and 120 DAE. For day 0, forage samples were immediately placed in freezer at −4 °C. For the first 14 DAE, the gas valve was released daily at 09:00, then for subsequent days, the valve was released every 7 d. At opening of each silo, two grab-samples were collected. One sample was frozen (−4 °C), and the other one was placed in a forced air oven at 60 °C for 48 h to determine DM content.

### 2.4. Determination of Nutritive Value

Frozen samples were placed on dry ice and sent for wet chemistry analysis at Dairy One Forage Laboratory (Ithaca, NY, USA). Prior to analysis, samples were thawed, dried in a forced air oven at 60 °C for 48 h, and ground to pass a 1 mm screen using a Wiley Mill (Thomas Scientific, Philadelphia, PA, USA). The following variables were determined: crude protein (CP, Method 990.03), pH (Method 973.41), DM (Method 930.15), and ammonia-N concentrations [12,13]. Concentration and volatile fatty acids scores (VFA) of acetic acid, propionic acid, butyric acid, isobutyric acid, and lactic acid were determined by a using packed column gas chromatography [14]. In brief, 50 g of sample was blended in 750 mL deionized water, and it was filtered. An aliquot of extract was mixed with 1:1 0.06M oxalic acid with 100 ppm trimethylacetic acid (internal standard). Samples were injected into a Clarus 680 Gas Chromatograph (Perkin Elmer, Waltham, MA, USA) with a 2 m × 2 mm Tight spec ID using 4% Carbowax 20M phase on 80/120 Carbopack B-DA (Sigma Aldrich, St. Louis, MO, USA). Lactic acid was determined using a 2700 Select Biochemistry Analyzer (YSI, Inc., Yellow Springs, OH, USA) that was equipped with an L-Lactate membrane [14]. Neutral detergent fiber (NDF), acid detergent fiber (ADF), and acid detergent lignin (ADL) concentrations were determined at the Auburn University Ruminant Nutrition Laboratory using methods that were described by Van Soest et al. [15]. All of the nutritive value parameters are reported on a DM basis based on respective DAE DM values.

### 2.5. Determination of In Situ Disappearance of DM

For the in situ DM disappearance, incubated samples were collected from the 45 DAE mini silos for Studies 1 and 2, and 28 DAE mini silos for Study 3. This approach was chosen due to the volume of samples to be incubated and availability of animals; while the adjustment in the ensiling period that was used reflected previous studies and collected data from Study 1 and 2 [5]. Two ruminally fistulated steers (*Bos* spp., ~600 kg body weight) were used. Steers were housed in individual stalls and fed wheat baleage ad libitum for a 30-d adaptation period. All of the procedures for the use of live vertebrate animals in experiments were approved by the Auburn University Institutional Animal Care 51 and Use Committee (2018-3244).

Samples were dried at 60 °C for 48 h and ground to pass a 1 mm screen using a Wiley Mill (Model 4, Thomas Scientific, Swedesboro, NJ, USA). One gram of ground material was placed in 5 × 10 cm nylon in situ bags (pore size 50 µm; ANKOM Technology, Macedon, NY, USA). Bags were heat-sealed and three replicates within forage mixture × inoculant strategy × steer combination were incubated for 0, 2, 4, 6, 12, 24, 48, and 72 h. Prior to their insertion, in situ bags were placed in hot water (39 °C) for 20 min, then they were placed inside polyester mesh bag and connected to a stainless-steel chain to ensure samples remained below the forage mat and inside the ruminal ventral sac [16]. All of the treatment bags (forage mixture × inoculant strategy × steer combination) were incubated and removed simultaneously at a given incubation time. Samples taken at 0 h were rinsed at 39 °C, and then, they were immediately placed in a freezer (−4 °C). At the end of incubation, all of the bags were placed in a plastic container, rinsed, and frozen (−4 °C). Prior to analysis being performed, bags were thawed and rinsed in agitating water bath (39 °C, 110 rotations per min for 5 min [17]). Then, they were individually rinsed with distilled water and dried at 60 °C for 48 h. Subsequently, samples were analyzed for NDF concentration determination and calculations [18].

### 2.6. Statistical Methods

Data from each study were analyzed using Proc GLIMMIX of SAS 9.4 [19]. For forage nutritive value data, a linear mixed model was used in which inoculant strategy; DAE and their interactions were considered to be fixed effects. Similarly, the in situ data were analyzed considering inoculant strategy and incubation period to be as fixed effects. Treatment means were separated using Fischer-protected least significant difference (LSD) test and reported for significant main effects and interactions (*p* ≤ 0.05).

## 3. Results

### 3.1. Botanical Composition

At the time of the harvest, the botanical composition was 36% wheat and 50% crimson clover for WC in study 1; it was 65% wheat and 25% T-raptor for WB in study 2, and it was 60% oats, 10% crimson clover, and 30% annual ryegrass for ROC in study 3. The remaining component percentages of the forage mass was comprised of weeds in all of the treatments.

### 3.2. Nutritive Value

Among the forage mixtures, the DM concentration was not different among the WC, WB, and ROC mixtures (Table 1). In study 1, there were no differences due to the inoculant strategy, the DAE, or their interactions. At 120 DAE, the CP, NDF, ADF, and lignin concentrations were 18.1, 53.2, 34, and 6.5% of the DM basis, respectively (Supplemental Table S1). The dry matter concentration was 49% less in the DAE 120 compared to DAE 0.

In study 2, the concentration of the DM decreased by 21% (*p* = 0.025) from 0 to 120 DAE (24.2 to 20.3% DM basis, respectively; Supplemental Table S2). The crude protein concentration was 6% greater (*p* = 0.031) under the IN than it was under the NIN (Table 2). Between the inoculant strategies, the NDF and ADF concentrations averaged a 52 and 30% DM basis, respectively (Supplemental Table S2). At 120 DAE, the NDF, ADF, and lignin concentrations were 542, 32.1, and 5.2% of the DM basis, respectively.

In study 3, the CP concentration was 11% greater (*p* = 0.022) for the NIN than it was for the IN strategy (16.6 vs. 15% DM basis, respectively) for the ROC mixture (Table 2). Between the inoculant strategies, the NDF and ADF concentrations averaged a 64 and 40% DM basis, respectively. Among the DAEs, the DM concentration decreased from 20 to 17% from 0 to 120 DAE (Table 3). The neutral detergent fiber concentration was 9% greater (*p* = 0.001) at 120 DAE than it was at 0 DAE. Similarly, the ADF concentration was 22% greater (*p* < 0.001) at 120 DAE than it was at 0 DAE. The concentration of lignin was 39% greater at 120 DAE than it was at 0 DAE (6.1 vs. 4.4% DM basis, respectively).

### 3.3. Fermentation Parameters

Between the inoculant strategies, the pH averaged 5.0, 5.0, and 5.5 for the WC, WB, and ROC mixtures, respectively (Table 4). In Study 1, the inoculant strategy affected the acetic (*p* < 0.001), lactic (*p* < 0.001), and propionic acid concentrations (*p* = 0.012) for the WC mixture. The acetic and propionic acid concentrations were doubles for the IN treatment versus the NIN treatment, while the lactic acid concentration was doubled in the NIN treatment versus the IN treatment. Among the DAEs, the pH decreased from 6.3 to 5.8 between 0 DAE and 120 DAE for the WC mixture (*p* < 0.001). The ammonia-N concentration was greater at 120 DAE (29.4% DM basis) than it was in the remainder of the DAEs. The lactic acid concentration was greater (*p* < 0.001) at 7 DAE (6.9% DM basis), and this dropped to a 1.7% DM basis at 120 DAE. The acetic (*p* < 0.001) and propionic acid (*p* = 0.033) concentrations were greater at 120 DAE (5.8 and 0.7% DM basis, respectively). The butyric acid concentration ranged from a 0.03 to a 2.5% DM basis between 0 DAE and 120 DAE. The ammonia concentration was greater (*p* < 0.001) at 120 DAE than it was over the remainder of the DAE times (5.1 vs. 0.0 to 60 DAE ranging from 0.8 to 1.5% DM basis).

Among the treatments of Study 2, the ammonia concentration averages a 0.7% DM basis (Table 4). The lactic (*p* < 0.001) and butyric (*p* = 0.080) acid concentrations doubled under the IN treatment versus that which resulted with the NIN treatment (Table 3) for the WB mixture. The propionic acid concentration (*p* = 0.021) was 20% greater for the IN treatment than it was for the NIN treatment. In Study 2, the pH decreased from 5.7 to 4.5 between 0 DAE and 120 DAE (*p* < 0.001). The ammonia concentration was greater (*p* = 0.012) at 120 DAE (0.9% DM basis). The butyric (*p* = 0.007) and acetic (*p* < 0.001) acid concentrations were greater at 120 DAE (0.5 and 6.5% DM basis). The lactic acid concentration was greater (*p* < 0.001) at 7, 14, and 21 DAE than it was for the remainder of the DAEs, including 120 DAE (5.4, 5.3, 4.9, and 2.3% DM basis, respectively). The propionic acid concentration ranged from a 0.80 to a 0.17% DM basis (*p* = 0.165). The ammonia N concentration was greater (*p* = 0.028) after 21 DAE, with it ranging from a 4.8 to a 6.2% DM basis, while the initial concentration of it was 2.3% of the DM basis.

In Study 3, there were no differences in the fermentation parameters due to the inoculant strategy for the ROC mixture (Table 4). The ammonia-N concentration averaged 24.7% of the DM basis. The pH decreased from 6.3 to 5.2 between 0 DAE and 120 DAE (*p* = 0.002). The acetic, propionic, and butyric acid concentrations were greater (*p* < 0.001) at 120 DAE (5, 1.1 and 5% DM basis, respectively). The lactic acid concentration was greater (*p* < 0.001) at 14 DAE than it was over the remainder of the times, including 120 DAE (4 vs. 0.03% DM basis). The ammonia and ammonia-N concentrations were greater (*p* < 0.001) at 120 DAE (6.2 and 37.1% DM basis, respectively).

### 3.4. Dry Matter Disappearance

**Figure 2:**
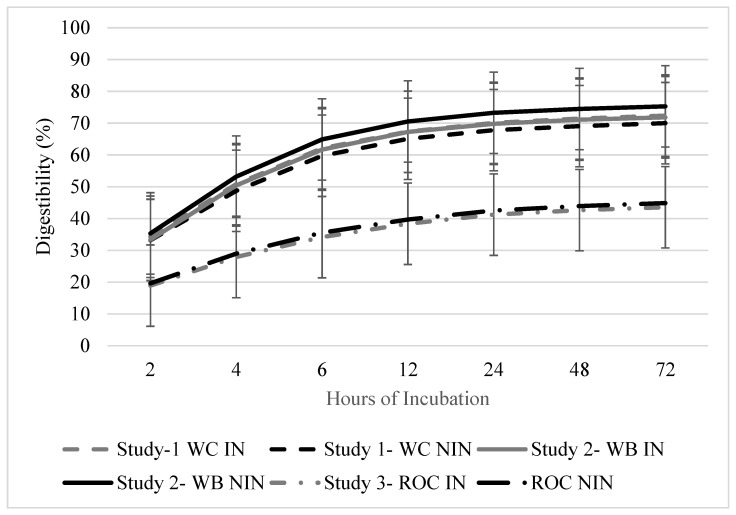
Cumulative digestibility of baleage from annual cool-season forage mixtures ensiled with or without inoculant after up to 72 h ruminal incubation. Study 1 included wheat and crimson clover with inoculant (WC-IN) and without inoculant (WC-NIN). Study 2 included wheat and brassica hybrid with inoculant (WB-IN) and without inoculant (WB-NIN). Study 3 included annual ryegrass, oat, and crimson clover with inoculant (ROC-IN) and without inoculant (ROC-NIN). Error bars indicate standard error of the mean (SEM).

The disappearance of the DM was greater in the initial 12 h for all of the studies (*p* < 0.010; Figure 2). In Study 1, there were no differences in the DM disappearance due to the inoculant strategy (*p* = 0.965) and DAE (*p* = 0.313). There were also no differences in the DM disappearance due to the inoculant strategy (*p* = 0.932) and the DAE (*p* = 0.320) for study 2. Studies 1 and 2 had similar in situ disappearance rates per hour throughout the 72 h collection period, which was probably due to presence of wheat in both of the forage mixtures (17.4, 25.8, 31.4, 34.3, 36.2, and 36.7% for 2, 4, 6, 12, 24, 48, and 72 h DM disappearance, respectively). In Study 3, the in situ disappearance was affected by the DAE (*p* < 0.001). The initial disappearance at 2 h was 20.0% and at 72 h incubation, and the disappearance value was 40.0%. The cumulative in situ disappearance values for the 72 h trial were 36.1, 37.1, and 40.0% for studies 1, 2, and 3, respectively.

## 4. Discussion

The 10-year average rainfall was 853 mm during the growing season (from September until April). During the experimental years, the annual precipitation averaged 531 and 1006 mm for studies 1 and 2 (2017/18) and study 3 (2018/19), respectively (Figure 1). In study 3, the limited rainfall between Feb and Mar decreased the forage production of the ROC mixture. The environmental conditions directly affected the plant growth and physiological responses, and this can impact the nutritive value. Comparatively, the ROC mixture had higher NDF and ADF concentrations than the WC and WB mixtures did. This was associated with the majority of the grasses in the composition of that mixture, but this can also be associated with the responses to the growing conditions.

The inoculant strategy had limited differences on the forage nutritive responses. Within the inoculant treatments, the DM concentration response for the WB mixture is most likely associated with there being a greater wheat proportion in the mixture (65%) than forb components. The CP concentration decreased by 11% in the ROC mixtures, and it increased by 6% in WB mixture with use of an inoculant. The concentrations of NDF and ADF were 50, 52, and 64% and a 30, 30, and 40% DM basis for the WC, WB, and ROC mixtures, respectively (Table 1). High-fiber concentrations in the ROC mixture were most likely due to 90% of its composition being made of grass components. In a 3-year study, Widenhoeft and Barton [20] evaluated the nutritive value of three *Brassica* spp. (rapeseed (*B. napus* L.), turnip (*B. rapa* L.), and turnip hybrid (*B. rapa* L. × *B. pekinensis* L.)) established under different sowing dates. The authors reported ADF and NDF concentrations ranging from 11 to 36% and 14 to 42%, respectively. In the current study, the WB mixture had greater ADF and NDF concentrations to the values that were reported by the authors. This is likely due to the forage in our study being more mature at the time of harvest compared that in Widenhoeft and Barton [20]. For the wheat-containing mixtures, the lignin concentration was 13% less in WB than it was in the WC mixture, which was most likely due to presence of a brassica hybrid that tends to accumulate less lignin at maturity when it is compared to the other forage species [21].

According to Kung et al. [7], the DM losses occur during the fermentation of high-moisture forages, which could lead to the occurrence of clostridial proliferation and protein degradation. Sears et al. [3] indicated that a 5% DM loss is expected throughout the ensiling process. At DAE 120, the DM concentration decreased by 51, 16, and 12% for the WC, WB, and ROC mixtures, respectively when they were compared to those at DAE 0. These ranges correspond to a decrease by up to 30% in the DM concentration over the 120 DAE period. The forage mixtures containing clovers were associated with a greater variation in the DM concentration from the initial to final DM concentrations, and they had greater ammonia-N concentrations at 120 DAE than the WB mixture did. In Study 1 and 2, the NDF, ADF, and lignin concentrations increased from 3 to 20% throughout the ensiling period (Supplemental Tables S1 and S2). Greater NDF and ADF concentrations at 120 DAE were expected due to the formation of fermentation end-products, and a resulting concentration of the refractory fibrous components [7].

Throughout the ensiling period, the WC mixture had a CP concentration ranging from 15 to 18%, while those for WB and ROC ranged from 12 to 14% and 15 to 17%, respectively (Supplemental Tables S1–S3). The presence of clover in the mixture increases the CP concentration [11], which was evident in the current study since WB had the lowest range. In a grazing study using the ROC mixture; Mason et al. [22] observed a 21.5% CP for the mixture. While Marchant et al. [23] reported a 19.5% CP for the wheat + annual ryegrass mixture under continuous stocking. Dillard et al. [24] found that mixtures of orchardgrass (*Dactylis glomerata* L.) with canola (*Brassica napus* L.), annual, rapeseed, and turnip had a ≤23% CP concentration in the brassica-containing diets. In the current study, most of the fermentation parameters were affected by the DAE due to transformation of the components throughout the fermentation process. For all of the forage mixtures, the pH decreased after 7 DAE, which is expected in order to stabilize of the forage material and maintain the nutritive value.

In conserved feedstuffs, ammonia production is associated with the degradation of plant proteins by the action of proteases and deaminases, whereas clostridial fermentation is associated with a high level of moisture and a pH that is above 4.8 [25]. Elevated ammonia concentrations are often associated with the decreases in the lactic acid bacteria and a delayed start in fermentation [25]. In our study, the ammonia concentrations for the clover-containing mixtures were greater, ranging from 4.3 to 6.3 and 1.6 to 6.2% of the DM basis from the initial start to 120 DAE, while for WB, this ranged from 0.3 to 0.9% of the DM basis. Conversely, the lactic acid concentrations ranged from 0.2 to 6.9, 0.01 to 6.3, and 0.03 to 5.4% of the DM basis for the WC, ROC, and WB mixtures, respectively. According to the work of Bolsen et al. [25], these responses are attributed to there being increased number of lactic acid bacteria during fermentation. Ammonia-N as a percent of the total N is a component of non-protein nitrogen (NPN) that is not as readily available to animals [26]. The general recommendation for the ammonia-N concentration is to have no more than 10 to 15% of the total N [7], which the ROC mixture exceeded at 7 DAE (16.3% DM basis, not shown). The WB mixture was the only forage mixture to remain below the recommended threshold by Kung et al. [7], with it having a range from 2.3 to 6.2% of the DM basis between 0 DAE and 120 DAE. The ammonia-N concentration for WC and ROC ranged from 4.74 to 29.4% and 10.9 to 37.1% of the DM basis, respectively.

In situ disappearance was greater in the first 12 h of the trial for all of the forage mixtures, which likely reflects a relatively low lag time and rapid initial digestion rate that is associated with these cool-season mixtures. The wheat-containing mixtures had similar values during the remaining intervals that were measured during the in situ disappearance trial, although the cumulative disappearance after 72 h was similar among all of the forage mixtures that were evaluated and this averaged 38%. Keim et al. [27] reported that the in situ DM disappearances of turnip and rapeseed were 92.6 and 89.3%, respectively [27]. Other studies have reported a forage wheat DM disappearance of 86.8%. In the WB mixture, in which the brassica hybrid was a small proportion of the forage mixture, the in situ DM disappearance was 73.6%. The slightly lower value than those which were obtained by previous studies is likely due to the low presence of brassica in the stand. Brassicas are reported to have over 90% in vitro fiber digestibility [1].

## 5. Conclusions

The effect of the microbial inoculant to improve the fermentation parameters and the nutritive value was inconsistent in the current study. This is in agreement with previous studies that also have found inconsistent results with microbial inoculants, and thereby warrants further study. The cumulative in situ disappearance rates were similar between the forage mixtures, which is probably due to there being a high proportion of grass component in all of the mixtures. The greater NDF and ADF that was observed in the current study when it was compared to previous studies reinforces the importance of harvesting at a proper maturity stage for optimizing the nutritive value of the conserved feed. However, even the increased fiber components of the nutritive value of these forages is adequate to meet the needs of all of the classes of beef cattle [28]. Based on our results, when they are properly managed, annual cool-season forage mixtures that are harvested and stored as baleage may provide a high-quality forage option to sustain animal growth and performance during periods of little to no forage growth (e.g., during the winter months). However, when poor fermentation occurs (i.e., Study 3), the forage nutritive value is actually reduced during the fermentation process. These factors combined can result in a poor animal performance.

## Figures and Tables

**Table 1:** Dry matter (DM), crude protein (CP), neutral detergent fiber (NDF), acid detergent fiber (ADF), and lignin concentrations of cool-season annual mixtures ensiled as baleage.

Response Variable	Forage Mixture					
	Study 1		Study 2		Study 3	
	WC ^1^	SEM ^2^	WB	SEM	ROC	SEM
DM (%)	18.9	0.58	22.4	0.37	18.5	0.29
CP (%)	17.1	0.55	13.4	0.25	15.8	0.42
NDF (%)	49.9	1.05	51.6	1.22	63.7	0.36
ADF (%)	30.2	0.56	29.5	0.51	39.9	0.26
ADL (%)	5.4	0.62	4.8	0.10	5.2	0.12

^1^ WC = wheat + clover; WB = wheat + brassica hybrid; ROC = ryegrass + oat + clover. ^2^ Standard error of the mean.

**Table 2:** Dry matter (DM), crude protein (CP), neutral detergent fiber (NDF), acid detergent fiber (ADF), and lignin concentrations under two inoculant strategies.

ResponseVariable	Forage Mixture								
	Study 1 ^1^			Study 2			Study 3		
	IN ^2^	NIN	SEM ^3^	IN	NIN	SEM	IN	NIN	SEM
DM (%)	18.3	17.9	0.58	21.7	23.0	0.37	18.4	18.6	0.29
CP (%)	17.8	16.4	0.55	13.8 ^a4^	13.0 ^b^	0.25	15.0 ^b^	16.6a	0.42
NDF (%)	50.1	49.7	1.05	52.0	51.2	1.22	63.8	63.5	0.36
ADF (%)	30.3	30.1	0.56	29.8	29.2	0.51	39.9	39.9	0.26
ADL (%)	5.3	5.4	0.35	4.9	4.8	0.10	5.2	5.1	0.12

^1^ Study 1 WC (wheat + clover); Study 2 = WB (wheat + brassica hybrid); Study 3 = ROC (annual ryegrass + oat + clover). ^2^ IN = microbial inoculant; NIN = No microbial inoculant. ^3^ Standard error of the mean. ^4^ In each row, means followed by different letters differ (*p* ≤ 0.05).

**Table 3:** Dry matter (DM), crude protein (CP), neutral detergent fiber (NDF), acid detergent fiber (ADF), and lignin concentrations of annual ryegrass + oat + crimson clover (ROC) mixture (Study 3) ensiled for 120 d.

Days After Ensiling	DM	CP	NDF	ADF	ADL
	%				
0	19.6	15.1	62.2 ^cde2^	36.2 ^e^	4.4 ^d^
7	18.1	16.1	65.1 ^b^	40.2 ^bc^	5.4 ^ab^
14	19.5	17.0	61.3 ^e^	38.4 ^d^	4.6 ^cd^
21	19.4	16.6	61.6 ^ed^	39.1 ^cd^	4.9 ^bcd^
28	18.3	15.4	64.0 ^bc^	40.1 ^bc^	5.3 ^bcd^
45	18.0	13.8	64.2 ^bc^	41.2 ^bc^	5.4 ^ab^
60	18.0	15.7	63.6 ^bcd^	40.8 ^bc^	5.6 ^ab^
120	17.3	16.7	67.5 ^a^	43.6 ^a^	6.1 ^a^
SEM ^1^	0.57	0.84	0.72	0.52	0.25

^1^ Standard error of the mean. ^2^ In each column, means followed by different letters differ (*p* ≤ 0.05).

**Table 4:** Fermentation parameters of forage mixtures ensiled under two inoculant strategies.

Response Variable	Forage Mixture											
	Study 1 ^1^				Study 2				Study 3			
	IN ^2^	NIN	SEM ^3^	*p-*Value	IN	NIN	SEM	*p-*Value	IN	NIN	SEM	*p-*Value
pH	4.9	4.9	0.12	0.705	4.5	4.6	0.04	0.024	5.5	5.4	0.08	0.491
Ammonia (crude protein equivalent %)	1.6	1.9	0.22	0.393	0.6	0.7	0.05	0.441	3.7	4.0	0.19	0.366
Ammonia-N (% of Total N)	8.9	11.0	1.50	0.311	4.5	5.1	0.39	0.292	25.2	24.2	1.57	0.651
Acetic acid (%)	5.7	2.2	0.38	<0.001	5.4	1.7	0.38	<0.001	3.2	3.5	0.26	0.510
Lactic acid (%)	2.6	5.3	0.41	<0.001	2.8	4.2	0.22	<0.001	0.9	1	0.16	0.641
Propionic acid (%)	0.4	0.2	0.07	0.012	0.1	0.1	0.09	0.021	0.5	0.4	0.05	0.485
Butyric acid (%)	0.3	0.9	0.33	0.221	0.1	0.2	0.04	0.008	2.5	2.4	0.11	0.981
Isobutyric acid (%)	0.05	0.02	0.026	0.446	0.01	0.01	0.002	0.400	0.20	0.20	0.16	0.951

^1^ WC = wheat + clover; WB = wheat + brassica hybrid; ROC = ryegrass + oat + clover. ^2^ IN = with inoculant; NIN = without inoculant. ^3^ Standard error of the mean.

## Data Availability

The data supporting reported results are available on request from the corresponding author.

## Supplementary Materials

The following supporting information can be downloaded at: https://www.mdpi.com/article/10.3390/ani12212929/s1, Table S1. Dry matter (DM), crude protein (CP), neutral detergent fiber (NDF), acid detergent fiber (ADF) and lignin concentrations of wheat and clover (WC) mixture (Study 1) ensiled for 120-d; Table S2. Dry matter (DM), crude protein (CP), neutral detergent fiber (NDF), acid detergent fiber (ADF) and lignin concentrations of wheat and brassica hybrid (WB) mixture (Study 2) ensiled for 120-d.
