# Supplementary material for: In Situ Ruminal Digestion, Fermentation Parameters, and Forage Nutritive Value of Cool-Season Baleage Ensiled under Contrasting Inoculant Strategies
Source: Animals (Basel). 2022 Oct 25;12(21):2929. doi: 10.3390/ani12212929 (PMC9655889; doi:10.3390/ani12212929)
Supplement: Supplementary file 1 [file animals-12-02929-s001.zip › animals-1784479-supplementary.pdf]

Table S1. Dry matter (DM), crude protein (CP), neutral detergent fiber (NDF), acid detergent fiber (ADF) and lignin concentrations of wheat and clover (WC) mixture (Study 1) ensiled for 120-d.

Days after ensiling	DM	NDF	ADF	ADL	CP
		- - - - - - - - - - - - - - - % - - - - - - - - - - - - - -			
0	20.9a[§]	51.7	30.3	5.5	15.4
7	20.6a	48.9	29.0	5.4	17.2
14	19.1ab	49.6	29.5	5.4	16.9
21	20.1a	49.5	29.5	5.6	17
28	18.4ab	48.5	29.2	4.9	19.1
45	16.9b	47.7	29.9	5.7	17
60	18.6ab	50.1	30.6	4.0	16.2
120	10.3c	53.2	33.8	6.5	18.1
SEM[‡]	0.95	0.04	1.08	0.68	1.07

[‡] Standard error of the mean. [§]Means followed by same letter in the column do not differ ($P \leq 0.05$).

Table S2. Dry matter (DM), crude protein (CP), neutral detergent fiber (NDF), acid detergent fiber (ADF) and lignin concentrations of wheat and brassica hybrid (WB) mixture (Study 2) ensiled for 120-d.

Days after ensiling	DM	NDF	ADF	ADL	CP
		- - - - - - - - - - - - - - - % - - - - - - - - - - - - - -			
0	24.2	49.5	28.3	4.8	12.2a§
7	22.9	52.8	29.8	4.9	13.4ab
14	22.7	51.8	29.0	4.9	13ab
21	22.1	54.1	30.3	5	13.3bc
28	22.5	49.1	28.3	4.5	13.9ab
45	22.5	51.2	29.6	4.7	13.7ab
60	21.5	49.9	28.9	4.7	13.8bc
120	20.3	54.2	32.1	5.2	14.2c
SEM[‡]	0.73	2.45	1.02	0.198	0.5

[‡] Standard error of the mean. [§]Means followed by same letter in the column do not differ ($P \leq 0.05$).
